# Inflammatory and Carcinogenic Biomarker Signatures in E-Cigarette Users: A Comprehensive Meta-Analysis of ∼24,000 Adults

**DOI:** 10.3389/ijph.2025.1608885

**Published:** 2025-11-12

**Authors:** Khalid A. Bin Abdulrahman, Amro K. Bin Abdulrahman, Razique Anwer

**Affiliations:** 1 Department of Medical Education, College of Medicine, Imam Mohammad Ibn Saud Islamic University (IMSIU), Riyadh, Saudi Arabia; 2 Department of Preventive Medicine, Public Health, and Lifestyle Medicine, Ministry of Health, Riyadh, Saudi Arabia; 3 Department of Pathology, College of Medicine, Imam Mohammad Ibn Saud Islamic University (IMSIU), Riyadh, Saudi Arabia

**Keywords:** electronic cigarette, e-cig, tobacco smokers, lung inflammation, lung cancer, carcinogenic biomarkers, meta-analysis

## Abstract

**Objectives:**

This meta-analysis examined the association between electronic cigarette (e-cig) use and biomarkers related to lung inflammation and carcinogenesis.

**Methods:**

A systematic review of PubMed, Scopus, Web of Science, EMBASE, and Cochrane Library (2014–April 2024) identified 16 studies including 24,079 adults. Biomarkers from urine, saliva, and plasma—cotinine, NNAL, NAT, and interleukins—were analyzed using one-way ANOVA and Tukey’s post-hoc tests. The GRADE framework assessed evidence certainty and risk of bias.

**Results:**

Among participants, 27.2% were smokers, 2.7% e-cig users, 66.0% non-smokers, and 4.1% dual users. E-cig users showed higher salivary cotinine than non-smokers (p = 0.033) but not smokers (p = 0.99). NNAL was significantly elevated in smokers (p = 0.035). E-cig users had increased inflammatory markers (IL-6, TNF-α) compared with non-smokers but lower than smokers. Carcinogenic biomarkers were present in e-cig users at reduced concentrations versus smokers. GRADE indicated low to high certainty with no or moderate bias.

**Conclusion:**

E-cigarette use is associated with biomarker alterations indicating inflammation and potential carcinogenesis, paralleling some effects of traditional smoking. Standardized longitudinal studies are needed to establish causality and long-term risks.

## Graphical Abstract

**Figure:**
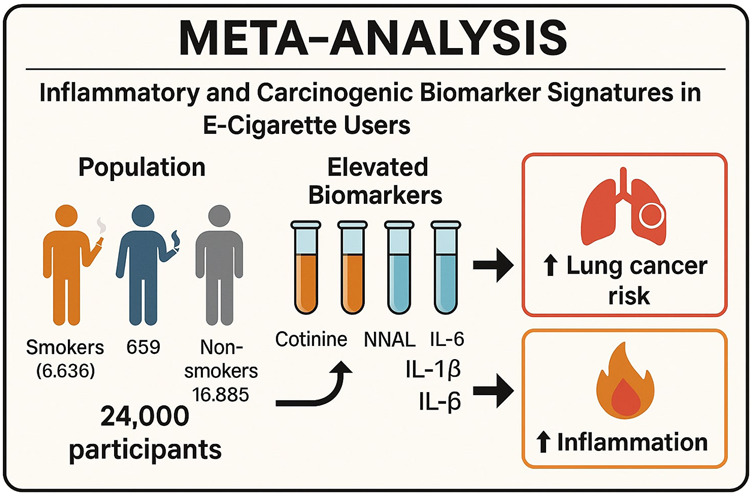

## Highlights

- Question What is the correlation between electronic cigarette consumption and the potential risks of lung cancer or lung inflammation?
- Findings The investigation revealed no statistically significant disparities in urinary cotinine concentrations between e-cig users and conventional smokers. Salivary cotinine levels exhibited differences between e-cig users and non-smokers but not between e-cig users and smokers. No substantial variations were observed in TSN levels. However, smokers demonstrated significant differences in NNAL levels compared to non-smokers. Interleukin (IL) markers in saliva and plasma showed no significant variations within or between groups, except elevated IL-8 levels in e-cig users.
- Meaning E-cig usage did not significantly impact biomarkers in urine, saliva, and plasma, analogous to traditional smoking. Nevertheless, both e-cig users and smokers exhibited higher levels of certain inflammatory markers compared to non-smokers, suggesting a potential increased risk of pulmonary inflammation and possibly LC. Further comprehensive research is imperative to elucidate the long-term health implications of e-cigs.

## Introduction

The global rise in the use of electronic vaping products (EVPs), including electronic cigarettes (e-cigs), has prompted growing public health concern [1]. Marketed as safer alternatives to traditional tobacco smoking, these devices deliver aerosols through battery-powered heating of e-liquids components (like nicotine, flavouring agents, propane-1,2-diol, propane-1,2,3-triol and water) [2]. Further, the pyrolysis decomposition of flavours at these high temperatures could produce a large number of unknown secondary chemical entities, raising the health risk of each flavour [3]. In addition, the pyrolysis process within a vaping device could transform vitamin E acetate (VEA) into ten different substances, including the highly toxic gas ketene, which is responsible for severe lung injuries [4]. While they are increasingly used by young adults and former smokers, the long-term health implications of e-cigs remain uncertain, particularly in relation to lung health and the development of lung cancer (LC) [5].

According to GLOBOCAN 2020 data, LC remains one of the most prevalent and deadly malignancies worldwide, accounting for over 2.2 million new cases and nearly 1.8 million deaths [6]. Although cigarette smoking is the established leading cause of LC, the emergence of e-cigs has introduced new challenges to the epidemiology of the disease [7]. Despite being viewed by some as less harmful, accumulating evidence suggests that e-cig aerosols may contain cytotoxic and carcinogenic compounds, including volatile organic compounds and tobacco-specific nitrosamines (TSNAs) [8]. These compounds have been implicated in promoting oxidative stress, DNA damage, and inflammatory responses in preclinical and human studies [7].

For instance, Shields et al. reported increased oxidative stress and DNA damage in murine lung cells following e-cig exposure, raising questions about their long-term carcinogenic potential [7]. Likewise, studies have found sustained levels of TSNAs—such as NNK (4-(methylnitrosamino)-1-(3-pyridyl)-1-butanone) and NNN (N-nitrosonornicotine)—in EVP aerosols, both of which are known contributors to LC pathogenesis. Urinary NNAL, the metabolite of NNK, has been widely validated as a biomarker of tobacco-related carcinogen exposure, but its utility in assessing e-cig exposure remains underexplored [8].

Although some investigations suggest e-cigs may be less harmful than conventional cigarettes, findings remain inconsistent, and data on systemic biomarkers—including inflammatory, oxidative, immune, and platelet activation markers—are limited in e-cig users. Furthermore, the absence of large-scale, long-term epidemiological studies complicates the assessment of their true health impact.

Given the increasing global prevalence of e-cig use, particularly among younger populations, and shifting tobacco control dynamics, it is imperative to better understand the potential associations between e-cig use and LC or lung inflammation. There is much current research focused on the issues of adolescent use of e-cig’s, nicotine addiction risks associated with vaping, general health risks, and unanswered questions about how effective e-cigarettes are as harm reduction tools for tobacco smokers [9].

This systematic review and meta-analysis aims to examine the current body of evidence regarding the relationship between e-cigarette use and the risk of developing lung cancer or lung inflammation. Addressing this gap is critical to informing public health strategies and regulatory frameworks that prioritize long-term respiratory health and reduce the global burden of LC.

## Methods

### Search Strategy

The present systematic review and meta-analysis were conducted following the guidelines outlined in the Preferred Reporting Items for Systematic Reviews and Meta-Analyses (PRISMA) statement and checklist [10].

A comprehensive review of the available literature was conducted, encompassing various electronic databases such as PubMed, Scopus, Web of Science, EMBASE, and Cochrane Library from 2014 to April 2024. Each database was tailored with specific keywords such as (“electronic cigarettes” OR “vaping” OR “e-cigarette”) AND (biomarker” OR “cotinine” OR “interleukin” OR “NNAL”) AND (“lung cancer,” AND “lung inflammation”). The areas of focus include electronic cigarettes, e-cigs, vapes, lung cancer, carcinogens, cotinine levels, and inflammatory markers. Additional investigations were conducted by scrutinizing the bibliographies of all retrieved articles from the databases.

### Eligibility Criteria

#### Inclusion Criteria

The study focused on human subjects who had used e-cigs or vapes and were diagnosed with LC (either by histological subtype or lesion location). The inclusion criteria for this analysis encompassed research articles, case reports, prospective or retrospective cohort studies, and observational studies. Only articles that specifically addressed saliva interleukins, carcinogen markers, and inflammatory markers of lung inflammation to e-cigs were selected for inclusion. All selected studies met the inclusion criteria for the meta-analysis.

#### Exclusion Criteria

Exclusions were implemented for correspondence, narrative, case series, published summaries, non-human investigations (including *in vitro* and *in vivo* studies), systematic reviews, meta-analyses, and review papers. The study excluded research on genetic factors, molecular mechanisms, the microbiome, colonization of lung bacteria, biomarkers, chemopreservation, clinical trials, Bayesian models, and computational models. Studies with inadequate data were also excluded from consideration.

### Data Extraction

Two authors independently conducted the process of extracting data from relevant studies. They used a predetermined template for data extraction, which included information such as the primary author, publication year, country, type of e-cig used, demographic details of the patient group, LC condition, size or stage of cancer, risk factors for LC or lung injury (such as smoking, alcohol consumption, and bacterial infection), other co-morbidities, and outcomes of interest. The data from selected studies was gathered using an Excel spreadsheet prepared in advance. Throughout the process, we alternated between data collection and assessment to ensure the results aligned with the analytical and interpretive framework.

### Study Flow

**FIGURE 1:**
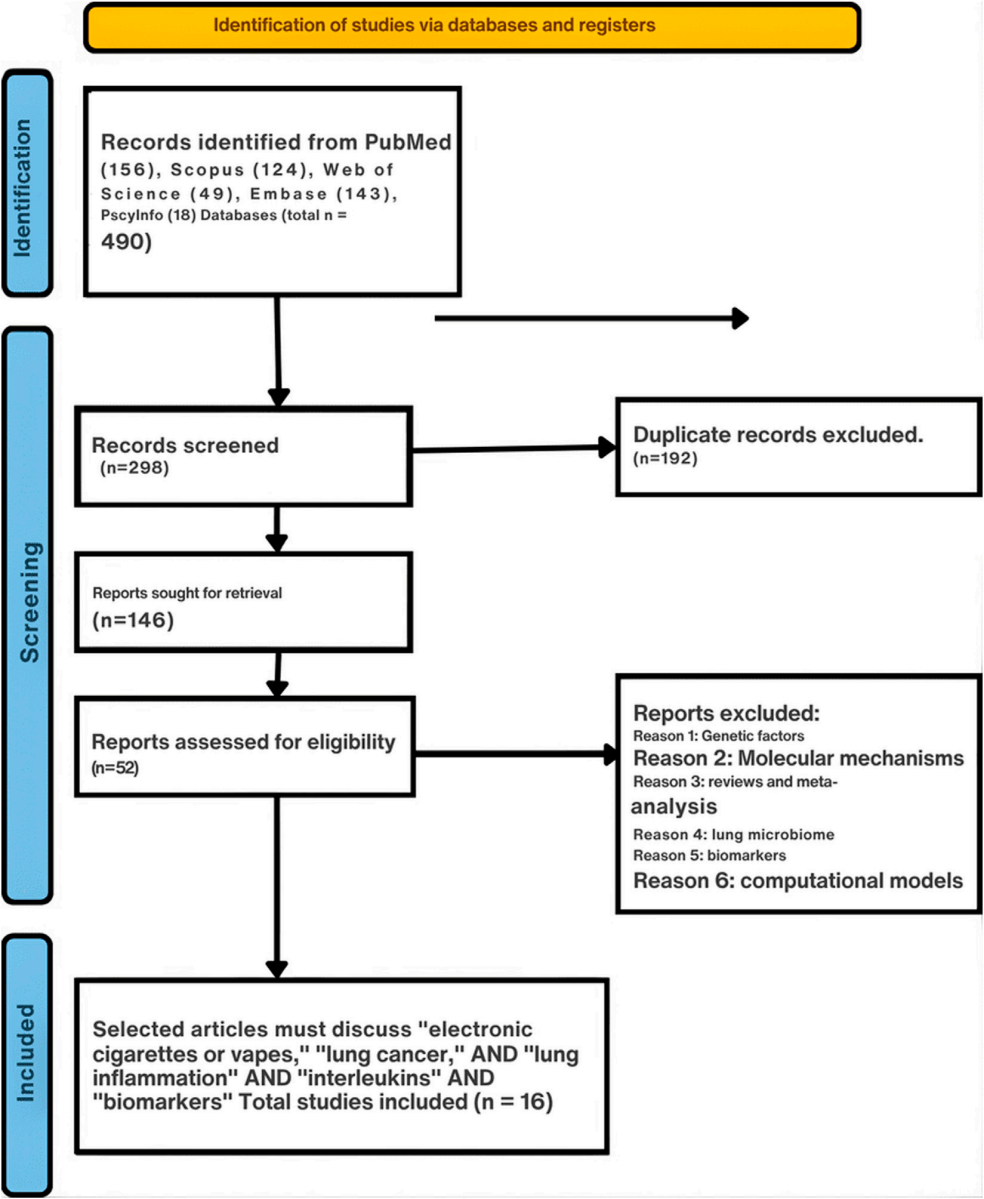
PRISMA flow diagram of included and excluded research studies (Multiple countries: United States, United Kingdom, South Korea, Canada, and Colombia; 2014–2024).

Figure 1 presents the incorporated and excluded research studies based on the parameters outlined in the PRISMA statement and checklist [10]. Following the initial exploration of electronic databases, the scrutiny of titles, abstracts, and full texts was conducted, leading to the selection of only a limited number of articles upon careful examination.

### Statistical Analysis

Statistical analysis was conducted using MedCalc software (version 9.5). Descriptive statistics were used to analyze the demographics and clinical characteristics of the subjects. Primarily, the focus was on e-cig usage and its impact on hematological, salivary, and urinary parameters, which are considered to have a direct influence on pulmonary health and increase the risk of cancer. The asymmetric distribution was estimated using the skewness and kurtosis methods. A standard one-way ANOVA was utilized to assess the mean variations among the groups, followed by Tukey’s post-hoc test for multiple comparisons at a 95% CI for urine, saliva, and serum cotinine levels. The NNAL levels and saliva interleukin markers between the groups were also assessed by one-way ANOVA.

A one-sample t-test was employed to evaluate the mean interleukin levels, predicated on a Gaussian distribution with a significance threshold set at 0.05. Additionally, an unpaired t-test incorporating Welch’s correction was utilized to juxtapose the average Interleukin levels between e-cig users and non-smokers. This parametric test, which does not presume equal standard deviations, was selected for the study. A two-tailed test with statistical significance set at p < 0.05 was used for the analysis. The forest plot was then generated by calculating the mean difference between the groups along with a 95% confidence interval.

### Publication Bias in Studies

The Newcastle - Ottawa Quality Assessment Scale was employed to assess the presence of publication bias in case studies. This evaluation focused on four key aspects: exposure, selection of the control group, determination of exposure, and assessment of the outcome [11]. The potential for publication bias was examined using the symmetry of the funnel plot and the Begg and Egger tests. Statistical significance was considered when P-values were below 0.05.

### GRADE Analysis

The risk of bias and certainty of evidence were assessed using the Grading of Recommendations, Assessment, Development and Evaluations (GRADE) [12]. The five major domains of GRADE method including risk of bias, imprecision, inconsistency, indirectness and publication bias were considered. We applied GRADE rules to rate evidence certainty levels for comparing cotinine level (plasma, saliva and urine), NNAL and NAT levels in urine, Saliva IN-6 and IL-1b, of smokers, non-smokers, e-cigratte users and dual users. Additionally, plasma interleukins were compared between smokers and e-cigratte users. The rating levels were high, moderate, low or very low. GRADE is a formal system for rating evidence quality and recommendation strength in systematic reviews, widely adopted globally to assess intervention impacts on resource use.

## Results

### Study Selection

Figure 1 presents the PRISMA flowchart. An initial database exploration identified 490 papers (156 from PubMed, 124 from Scopus, 49 from Web of Science, 143 from Embase, and 18 from PscyInfo). After removing duplicates, 192 papers met our elimination criteria. Subsequently, 52 articles were evaluated for eligibility, while 94 were rejected as they discussed other topics such as genetic influences, lung microbiomes, computational blueprints, and molecular processes. Of the 52 papers evaluated, 36 were excluded since they discussed reviews and meta-analyses, cancer broadly, therapeutic facets, and unrelated malignancies. Consequently, 16 papers were deemed suitable and incorporated into the analysis [13–28].

### Study Characteristics

**FIGURE 2:**
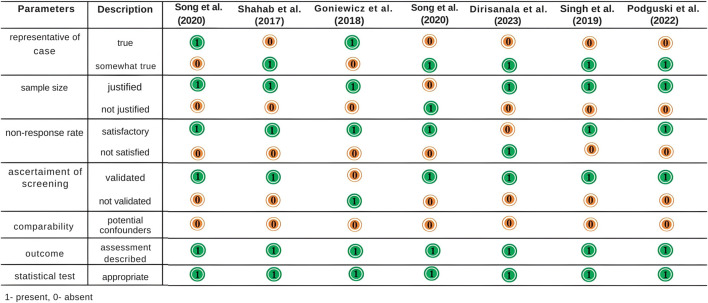
Newcastle–Ottawa scale scores for quality assessment of selected studies (Multiple countries: United States, United Kingdom, South Korea, Canada, and Colombia; 2014–2024).

Sixteen articles met the inclusion criteria for the meta-analysis [13–28]. Among the 16 articles, 12 originated from the USA, and one each from the UK, South Korea, Canada, and Colombia. Eleven of these studies were cross-sectional, encompassing both prospective and observational research designs. Four were randomized control cross-sectional pilot studies, and one was a scientific report. Table 1 presents the demographic characteristics of the selected studies. According to the Newcastle-Ottawa scale, the chosen articles scored above 7 points, signifying high-quality research (Figure 2).

**TABLE 1:** Characteristics of studies included in the meta-analysis (Multiple countries: United States, United Kingdom, South Korea, Canada, and Colombia; 2014–2024).

Groups	Parameters	[24]	[14]	[18]	[25]	[22]	[27]	[17]	[23]	[15]	[19]	[26]	[21]	[16, 16]	[28]	[13]	[20]
	Place	New York	UK	USA	USA	USA	USA	USA	USA	USA	South Korea	USA	Canada	USA	USA	USA	Columbus
	Type of Study	Cross-sectional study	Cross-sectional study	Cross-sectional study	Randomized control pilot study	Randomized control pil0t study	Pilot cross-sectional study	Cross-sectional study	Cross-sectional study	Cross-sectional observational study	Cross-sectional study	Pilot cross-sectional study	Cross-sectional study	Cross-sectional study	Scientific report	Prospective cross-sectional study	Cross-sectional study
Smoker	No. of participants	16	37	2,411	-	-	-	20	18	39	2,627	12	-	-	1,341	14	16
	Mean age	26	34.4	36.5	-	-	-	41.9	51.66	42.4	44	40.25	-	-	49.5	30.71	26
	No. of Male	12	21	1,059	-	-	-	8	-	39	2,229	5	-	-	642	6	12
	No. of Female	4	16	1,352	-	-	-	12	-	0	398	7	-	-	698	8	4
	smoking/day	16	13.9	15.4	-	-	-	15.9	-	16.2	14.1	-	-	-	-	11.8	16
	No. of years of smoking	6.6	17.8	7	-	-	-	26	-	17.2	-	-	-	-	31.3	-	8
e-cigarette	No. of participants	15	36	247	15	22	6	20	15	37	44	12	-	7	151	12	15
	Mean age	27	38.5	36.5	25	35.54	22.3	31.3	51.66	28.3	38.2	34.92	-	40.4	41.2	26.33	27
	No. of Male	10	29	98	7	10	3	12	-	37	35	10	-	4	82	5	10
	No. of Female	5	7	149	8	12	3	8	-	0	9	2	-	3	69	8	5
	Smoking/day	10.9	15.9	1.3	-	7.75	3	18.7	-	9.2	-		-	-	-	-	13
	No. of years of smoking	2.7	21.9	7	-	2	2.5	1.2	-	3.1	-		-	-	6.2	-	8
Non-smoker	No. of participants	42	-	1,655	15	26	6	19	15	38	12,182	12	-	10	1846	13	12
	Mean age	25	-	36.5	27	33.88	22.3	40.6	51.66	40.6	48	35.67	-	40.2	49.5	30.38	26
	No. of Male	17	-	607	5	11	3	5	-	38	4,125	2	-	5	784	7	5
	No. of Female	25	-	1,048	10	15	3	14	-	0	8,057	10	-	5	1,062	5	7
	Smoking/day	0	-	-	-	-	-	-	-	-	-	-	-	-		-	-
	No. of years of smoking	0	-	-	-	-	-	-	-	-	-	-	-	-		-	-
Dual	No. of participants		-	792	-	-	-	-	16	-	-	12	48	-	115	-	-
	Mean age		-	18.55	-	-	-	-	51.66	-	-	39.42	35.9	-	40	-	-
	No. of Male		-	283	-	-	-	-	-	-	-	7	34	-	37	-	-
	No. of Female		-	509	-	-	-	-	-	-	-	5	14	-	73	-	-

- not available.

Data collected from 16 studies encompassed a total of 24,079 individuals; among these, 27.21% (n = 6,551) were conventional smokers, 2.72% (n = 654) utilized e-cigs, 66.00% (n = 15,891) were non-smokers, and 4.08% (n = 983) were dual users. Statistical evaluations revealed an asymmetric distribution (skewness >1.45) with a kurtosis value exceeding 4.0, denoting a Laplacian distribution for e-cig users, non-smokers, and dual users. Conversely, the smoker group’s data exhibited a kurtosis value of 0.5 and skewness of 1.48, indicative of a positively skewed distribution.

The mean ages were determined as follows: 38.48 ± 8.44 years for smokers, 33.61 ± 7.72 years for e-cig users, 36.24 ± 9.26 years for non-smokers, and 37.11 ± 11.27 years for dual users. The majority of traditional smokers (61.18%) and e-cig user users (53.82%) were males, while females constituted the majority of non-smokers and dual users (64.57% and 61.14%, respectively). On average, traditional smokers consumed 14.91 ± 1.49 cigarettes daily, whereas e-cig users used an average of 9.97 ± 5.78 e-cigs per day. Based on the gathered literature, individuals who smoke have typically used conventional cigarettes for an average of 16.27 ± 9.38 years. In comparison, their use of e-cigs spans approximately 6.07 ± 6.22 years.

### Comparison of Urine, Saliva, and Serum Cotinine Levels Between the Groups

Five articles revealed no significant differences in UCot levels between the groups (p > 0.05) (Table 2) [14, 17–19, 21]. Specifically, comparisons showed no marked differences between e-cig users and non-smokers (p = 0.998), e-cig users and traditional smokers (p = 0.684), and e-cig users and dual users (p = 0.647). However, the selected articles inconsistenly reported the dose-response data for the UCot levels with nicotine concentration in e-liquid. In contrast, cotinine concentrations in saliva, as reported in two articles, exhibited significant divergence between e-cig users and traditional smokers (p < 0.05) [14, 15]. A notable distinction was found between e-cig users and non-smokers (p = 0.033). However, no significant differences were detected between e-cig users and traditional smokers (p = 0.99), nor between e-cig users and dual users (p = 0.928). Plasma cotinine data was exclusively available for individuals using e-cigs and those who do not smoke. The analysis excluded smokers, as only one study provided relevant data [13]. Significant variation was observed for e-cig users (p = 0.036); non-smokers did not exhibit significant variation (p = 0.382). A chi-square test indicated notable differences between e-cig users and non-smokers (p = 0.0441).

**TABLE 2:** Comparison of cotinine, 4-(methylnitrosamino)-1-(3-pyridyl)-1-butanol, N′-nitrosoanatabine, and interleukin levels in urine and saliva among groups (Multiple countries: United States, United Kingdom, South Korea, Canada, and Colombia; 2014–2024).

Sample	Statistical analysis	e-cigarette	Smoker	Non-smoker	Dual
Urine cotinine	Mean (ng/mL)	175.87	490.19	133.70	559.74
	SD	315.58	560.34	266.34	588.53
	t-value	1.25	1.96	1.00	1.65
	P value	0.28	0.12	0.39	0.24
	95% CI	−215.98 to 567.73	−265.56 to 1,185.94	−290.10 to 557.51	−902.25 to 2021.73
Saliva cotinine	Mean (ng/mL)	193.81	188.33	1.43	224.08
	SD	24.07	53.79	1.23	105.90
	t-value	13.94	6.06	1.64	2.99
	P value	0.0051^a^	0.0261^a^	0.3480	0.2053
	95% CI	134.01 to 253.60	54.72 to 321.95	−9.62 to 12.48	−727.41 to 1,175.59
Plasma cotinine	Mean (ng/mL)	141.73	-	19.51	-
	SD	48.24	-	30.44	-
	t-value	5.089	-	1.110	-
	P value	0.036^a^	-	0.382	-
	95% CI	21.90 to 261.56	-	−56.12 to 95.14	-
Urine NNAL	Mean (pg/mg)	7.93	169.70	5.42	132.60
	SD	7.89	120.50	9.57	113.70
	t-value	2.246	3.148	1.132	2.332
	P value	0.088	0.035^a^	0.339	0.102
Urine NAT	Mean (pg/mg)	2.85	64.43	2.92	78.85
	SD	1.50	44.73	-	67.95
	t-value	2.69	2.04	-	1.64
	P value	0.227	0.291	-	0.348
Saliva IL-6	Mean (pg/mg)	6,007	52,501	3,339	1.65
	SD	10,386	74,245	5,769	-
	t-value	1.002	1.00	1.003	-
	P value	0.422	0.5	0.422	-
Saliva IL-1β	Mean (pg/mg)	5,071	36,784	2084	84.53
	SD	9,953	63,407	3,946	117.8
	t-value	1.019	1.005	1.056	1.015
	P value	0.383	0.421	0.368	0.495

^a^ Statistically significant; - not available; for uniformity purposes, nM of cotinine was converted in some articles to ng using an online cotinine unit conversion calculator; NNAL- 4-(methylnitrosamino)-1-(3-pyridyl)-1-butanol; NAT- N′-nitrosoanatabine; IL- Interleukin.

### Comparison of NNAL Level Between the Groups

Five studies examined NNAL concentrations in urine [13, 14, 18, 21, 28]. No statistical differences were found among e-cig users, non-smokers, and dual users (p > 0.05), whereas the smoker group exhibited significant variations (p = 0.0346) (Table 2). In multiple comparisons, a notable difference was observed between e-cig users and smokers (p = 0.037). In contrast, comparisons between e-cig users and non-smokers or dual users were not significant (p > 0.05). The mean NAT concentration showed no statistical variation (p > 0.05) within the groups (the non-smoker group was excluded since only one measurement was available) [14, 18]. Chi-square tests among the groups also indicated no significant difference in urinary NAT levels.

### Comparison of Saliva Interleukin Markers

Three studies examined the impact of e-cig usage on saliva IL-6 concentrations [15, 22, 23], while four studies focused on saliva IL-1β levels [15, 22, 23, 26]. For consistent analysis, the interleukin measurements were standardized to pg/mL. The mean concentration of saliva IL-6 and IL-1 β showed high levels in smokers, followed by e-cig users compared to non-smokers. However, IL-6 and IL-1β in saliva showed no significant differences either within individual groups or between different groups (p > 0.05) (Table 2).

### Comparison of Plasma Interleukin Markers

**FIGURE 3:**
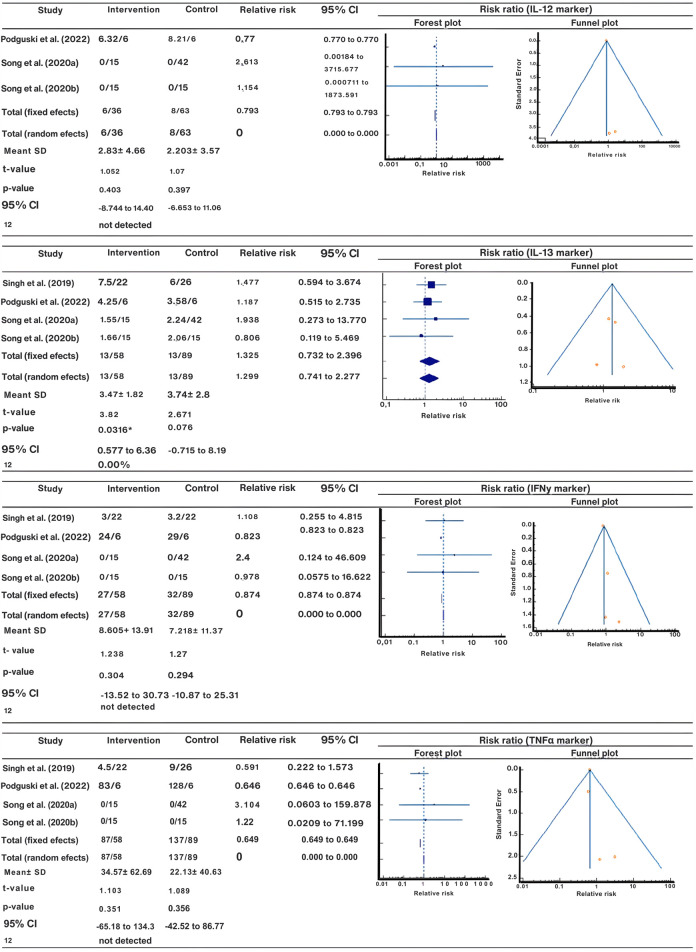
Forest and volcano plots of plasma interleukin levels (Multiple countries: United States, United Kingdom, South Korea, Canada, and Colombia; 2014–2024).

**FIGURE 4:**
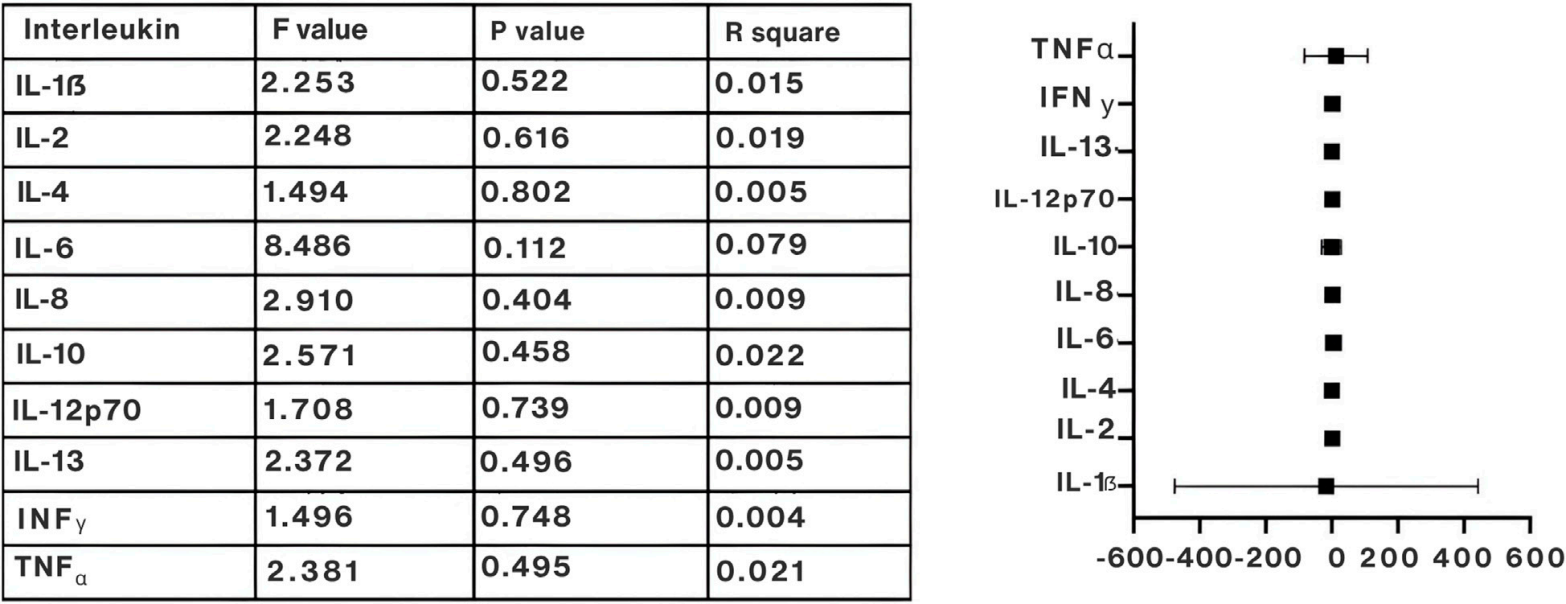
Forest plot comparing plasma interleukin levels between electronic cigarette users and non-smokers (Multiple countries: United States, United Kingdom, South Korea, Canada, and Colombia; 2014–2024)

The impact of e-cig consumption on plasma interleukin concentrations was documented in three to four scholarly articles. Notably, comparisons were exclusively made between e-cig users and individuals who do not smoke, thereby excluding smokers and those who use both e-cigs and traditional cigarettes from the analysis. Based on the evaluated data, there was no statistically significant difference (p > 0.05) in interleukin levels within the e-cig cohort except for IL-8 (p = 0.028). Similarly, there were no notable variations for the non-smoker cohort except for IL-13, which demonstrated a significant alteration (p = 0.0316) (Figure 3; Supplementary Table S1). E-cigarette users exhibited higher mean concentrations in certain inflammatory markers compared to non-smokers, although these differences did not consistently reach statistical significance. Figure 4 elucidates the statistical comparison of interleukin levels between the groups. The forest plot illustrates the mean plasma interleukin concentration and 95% confidence intervals. The presented data showed no significant differences between the groups (p > 0.05). Our findings indicate that e-cig use does not impact plasma interleukin levels.

### GRADE-Based Analysis of Biomarker Outcomes in E-Cigarette Users

The selected articles were further assessed for GRADE-based synthesis among the biomarkers used in the study. The evaluated biomarkers included cotinine (in urine, plasma, and saliva), tobacco-specific nitrosamines (NNAL and NAT), and inflammatory markers (IL-6 and IL-1β) in saliva (Supplementary Table S2).

Five studies on urine cotinine showed moderate bias risk due to population size variations and omission of certain groups. While two studies showed inconsistency due to variations in smokers, all reported no indirectness, imprecision, or publication bias, with evidence certainty ranging from low to high. For plasma cotinine, three studies showed no or moderate bias risk, though two lacked proper smoker representation, and one showed moderate indirectness due to nasal sampling. Despite moderate inconsistency, these maintained high certainty. Three saliva cotinine studies demonstrated moderate bias risk and inconsistency due to incomplete comparator groups, but showed no indirectness or imprecision with high certainty. Five studies reported urinary NNAL levels, with three showing no bias and two having moderate bias due to missing groups. All studies demonstrated high certainty despite some inconsistency. Two urine NAT studies showed high certainty, with one showing moderate inconsistency and bias. For inflammatory biomarkers, three studies evaluated salivary IL-6 levels. One had no bias but moderate indirectness due to gingival health focus, while others showed low-to-moderate bias with large variations. Salivary IL-1β was assessed in four studies, with varying bias levels and indirectness due to periodontal assessments. Certainty ranged from low to high across all studies.

### GRADE Evidence Profile for Inflammatory Biomarkers

A comprehensive GRADE assessment was conducted for studies evaluating the impact of e-cig use on pro-inflammatory and anti-inflammatory biomarkers in adults. Across all biomarkers analyzed—including IL-1β, IL-2, IL-4, IL-6, IL-8, IL-10, IL-12p70, IL-13, IFN-γ, and TNF-α—the overall risk of bias was consistently rated as moderate, primarily due to variation in population size across studies. No studies exhibited serious concerns regarding indirectness or imprecision, and publication bias was not detected in any of the reviewed data (Supplementary Table S3).

IL-1β, IL-6, IL-8, IL-10, IL-13, IFN-γ, and TNF-α showed moderate to high certainty across studies, with some variability in effect sizes noted in Singh et al. [22]. IL-1β and IL-8 levels showed inconsistent findings in some studies, while Podguski et al. [27] and Song et al. [25] reported high certainty. IL-2 and IL-12p70 demonstrated high certainty across all studies, showing consistent results and no imprecision. IL-4 and IL-13 showed moderate to high certainty, with Podguski et al. [27] reporting moderate certainty due to variability, while Song et al. [24] maintained high certainty. IFN-γ showed moderate certainty in Singh et al. [22], but high certainty in other studies. These findings indicate a consistent association between e-cigarette use and elevated inflammatory cytokines, suggesting links to lung inflammation and carcinogenesis. Despite some heterogeneity, the evidence supports high confidence in the observed biomarker changes.

## Discussion

This meta-analysis of 16 high-quality studies involving over 24,000 adults provides a comprehensive evaluation of inflammatory and carcinogenic biomarker profiles among e-cigarette users compared to conventional smokers, dual users, and non-smokers. Our findings show mixed evidence regarding nicotine exposure, tobacco-specific nitrosamine (TSNA) levels, and inflammatory cytokine profiles, raising important considerations for understanding the long-term health risks of e-cigarette use.

### Cotinine Biomarker Findings

This meta-analysis study offers novel insights into e-cig products containing cotinine when used by adult smokers, from various geographic regions (like USA, UK, South Korea, Canada and Columbia). Cotinine, the primary metabolite of nicotine, is detectable in plasma, urine, and saliva. Evaluating cotinine levels in urine, saliva, and plasma is an effective method to gauge tobacco exposure, as cotinine in an unstimulated total salivary flow rate has a relatively long half-life [15, 29]. As a biomarker, cotinine is involved in assessing nicotine and e-cig use [30]. In this study, tobacco smokers, e-cig users, dual users, and non-smokers were screened based on our inclusion criteria.

The current findings indicated that UCot levels were significantly higher among smokers (490.19 ± 560.34 ng/mL), e-cig users (175.87 ± 315.58 ng/mL), and dual smokers (559.74 ± 588.53 ng/mL) compared to non-smokers (133.70 ± 266.34 ng/mL). Behera et al. [31] documented UCot values with a mean of 2,736.20 ± 983.29 ng/mL. Passive smokers exhibited cotinine levels of 285.75 ± 86.30 ng/mL, markedly higher than non-smokers (7.30 ± 2.47 ng/mL). Our observations revealed that smokers had a mean salivary cotinine level of 188.33 ± 53.79 ng/mL. Dual smokers showed a mean value of 224.08 ± 105.90 ng/mL, non-smokers had a mean value of 1.43 ± 1.23 ng/mL, and e-cig users had 193.81 ± 24.07 ng/mL. This meta-analysis effectively differentiates between non-smokers, e-cig users, and tobacco smokers, aligning with the data presented by Sharma et al. [29].

Most of the research on cotinine levels in e-cig users has focused on plasma and saliva measurements. Göney et al. [32] conducted a pioneering study on UCot levels in e-cig users in Turkey. This study revealed a positive correlation between UCot levels and nicotine concentration in e-cig liquids [32]. The median level of UCot in regular e-cig users was significantly higher than in the non-smoking group (276.11 [95% CI: 58.01–284.15] ng/mL vs. 5.21 [95% CI: 4.65–23.72] ng/mL, *P* < 0.001). Factors that affect UCot levels in e-cig users included age (P = 0.041), nicotine concentration in the e-cig liquid (P = 0.013), and flavor of the e-cig liquid (e.g., menthol or non-menthol; P = 0.040) [30]. E-cigs and traditional cigarettes produced similar effects on serum cotinine levels after both active (60.6 ± 34.3 vs. 61.3 ± 36.6 ng/mL) and passive (2.4 ± 0.9 vs. 2.6 ± 0.6 ng/mL) smoking [33]. It was found that e-cigs cause fewer changes in lung function but have a comparable nicotinic impact to traditional cigarettes [33].

### Carcinogenic Biomarkers (NNAL, NAT) Findings

Furthermore, NNAL has been extensively used as a biomarker to evaluate human exposure to NNK via its urinary metabolite [8]. Xia et al. [34] found that NNAL levels were 64.5 and 3.7 times higher in tobacco and e-cig users (p-value = 0.0001). In the Population Assessment of Tobacco and Health (PATH) study, e-cig users had an NNAL of 6.3 ng/g creatinine (95% CI: 4.7–7.9) [35]. In our study, urine NNAL was approximately 7.93 pg/mg, which was 31.65 times higher compared to non-smokers. Significantly lower levels of TSNA in e-cig users compared to other tobacco users align with lower levels of TSNA in e-liquids compared to cigarette smoke.^32^ In general, daily exclusive e-cig users have higher NNN levels (5.2 ng/g creatinine) compared to NAT levels (4.5 ng/g creatinine). While TSNA users tend to have lower NNN levels than NAT, e-cig users have higher NNN levels. One possible explanation for this difference is that e-cig users may generate fewer nitrosation of tobacco alkaloids compared to users of other products [34]. The presence of NNK in rodents induces tumors in the lung, nasal mucosa, pancreas, and liver and has been linked to LC in smokers [36]. Moreover, NNAL, the primary metabolite of NNK, has similar carcinogenic properties as NNK [37]. According to some reports, electronic nicotine delivery devices may affect TSNA exposures due to changing tobacco usage patterns [38, 39].

### Inflammatory Biomarkers

Another primary objective of this study was to assess the impact of e-cig vaping, cigarette smoking, and dual-use on biomarkers related to inflammation. The selected biomarkers (with elevated IL-6, IL-8, TNF-α, and IL-1β) in our study are associated with systemic inflammation, oxidative stress, tissue injury/repair, and angiogenesis. Whereas direct pathological equivalence was not reported, our comparative data suggest the similarity in cytokine elevations in inflammation [40, 41]. Cigarette smoke triggers oxidative stress and inflammation of the lungs, leading to COPD [22]. This oxidative stress from cigarette smoke activates the inflammatory response by increasing cytokines such as IL-6 and IL-8 [42]. A study by BinShabaib et al. [43] noticed no significant difference in the pro-inflammatory cytokines markers (IL-1β, IL-6, IFN-γ, TNF-α, and MMP-8) in e-cig users compared to non-smokers. However, Al-Aali et al. [44] used peri-implant sulcular fluid (PISF) to compare 47 e-cig users with 45 non-smokers, revealing statistically significant increases in TNF-α and IL-1β levels in e-cig users [44]. Our findings align with several studies showing elevated levels of IL-2, IL-4, IL-6, IL-8, TNF-α, IL-1β, and IFN-γ in e-cig users, although no significant differences were observed compared to non-smokers [22, 45]. Prolonged oxidative exposure can lead to various diseases, including cardiovascular disease, lung fibrosis, and cancers of the blood and lungs [46].

Furthermore, e-cig users exhibited significantly elevated levels of IL-2, IL-6, TNF-α, and INF-γ, alongside reduced levels of IL-10, compared to non-smokers. Similarly, traditional smokers showed increased levels of IL-2, IL-6, TNF-α, and INF-γ and decreased levels of IL-10 compared to non-smokers. In particular, while both e-cigs and conventional cigarettes were linked to increased inflammation via meta-transcriptomics, distinct pathways that mediate these effects were identified [23]. It was reported that the pro-inflammatory factor IL-8 can inhibit cell growth in normal cells but can promote cell division and invasion and alter tumor suppression in cancerous cells by comprehending the nuclear factor- ĸB pathway among tobacco smokers [47].

### Biological Implications for Carcinogenesis

This meta-analysis shows a significant link between e-cigarette use and elevated biomarkers for lung inflammation and cancer risk. However, it is unclear whether existing lung cancer prediction models developed for Western populations are directly applicable to Asia due to cultural and socioeconomic differences, including varying healthcare systems, smoking habits, environmental exposures, and genetic factors [48, 49]. Elevated inflammatory cytokines like IL-6 and IL-1β were observed, indicating that e-cigarette aerosol triggers inflammatory responses in respiratory tissues [24, 25, 50]. Cotinine levels confirmed systemic nicotine absorption, while tobacco-specific nitrosamines (NNAL and NAT) suggest measurable carcinogenic risks [51, 52]. Despite some variability and study limitations, the evidence certainty remained high for most biomarkers. These findings highlight a critical biological plausibility that e-cigarettes contribute to early pathological changes associated with pulmonary diseases and malignancy [53].

### Strengths and Limitations

The strengths of this meta-analysis include the large pooled sample size (∼24,000 participants, from multinational countries like USA, UK, South Korea, Canada, and Colombia), the integration of multiple biomarker types (nicotine metabolites, TSNAs, and cytokines), and the application of GRADE methodology to assess the certainty of evidence. However, several limitations have been noted. First, most included studies were cross-sectional, limiting causal inference and temporal associations. Second, heterogeneity across study designs, populations, and e-cigarette devices may have contributed to variance in effect sizes. Moreover, dose of smoked tobacco or e-liquid data is inconsistent in the selected articles. Third, many biomarker analyses lacked representation from dual users or had underrepresentation of smokers or non-smokers as reference groups, which may have reduced comparative power (especially cytokine data). Fourth, in GRADE based meta-analysis, moderate risk of bias was noticed in few articles, due to the inconsistencies occurred in the outcomes. This limited high quality longitudinal studies and lack of standardized biomarker thresholds, which restricted the interpretations. Finally, long-term outcomes could not be directly assessed, highlighting the need for longitudinal and mechanistic follow-up studies.

### Public Health and Clinical Relevance

These findings carry significant implications for regulatory science and clinical guidance. On one hand, the reduced NNAL levels in e-cigarette users compared with smokers support the argument for relative harm reduction, particularly in smoking cessation contexts. On the other hand, the consistent evidence of nicotine dependence and elevated inflammatory pathways suggests that e-cigarettes are not biologically inert and may contribute to chronic disease risk. Clinicians and policymakers should recognize this duality: while e-cigarettes may mitigate some harms compared with combustible smoking, their use cannot be equated with non-smoking safety. Preventive health strategies should therefore remain cautious, especially concerning initiation among adolescents and non-smokers, where long-term biomarker alterations may predispose to malignancy and cardiopulmonary disease.

### Conclusion

This meta-analysis suggests that whole salivary cotinine levels exhibit significant differences between tobacco and e-cig smokers. However, UCot levels did not show considerable variation among the groups studied. When evaluating TSN carcinogens, including urine NNAL and NAT, the smoker group demonstrated significantly higher variations compared to e-cig, non-, and dual smokers. Whole salivary IL-1β and IL-6 were elevated in traditional cigarettes and e-cigs smokers relative to non-smokers and dual users, indicating adverse effects from inflammation and oxidative stress. Although plasma interleukin data did not reveal statistically significant differences between the groups, interleukin marker levels were higher in e-cig users than in non-smokers.

This research indicates that the use of e-cigs negatively impacts oxidative stress and inflammatory responses, leading to tissue remodeling. The continuous activation of these mediators may contribute to the development of cardiovascular and pulmonary diseases. Certain mediators identified in various biological fluids could serve as vital noninvasive biomarkers for e-cig vaping. These biomarkers are valuable for the evaluation of lung injuries related to e-cig smoking and play a crucial role in regulatory and diagnostic processes associated with vaping in humans. This meta-analysis shows e-cig use correlates with elevated biomarkers of lung inflammation and carcinogenic risk, including cotinine, NNAL, IL-6, and IL-1β. While evidence certainty varied, findings indicate e-cigs may cause biological changes linked to lung disease, supporting the need for research, regulation, and public health interventions.

### Summary

This meta-analysis examined the association between e-cigarette use and biomarkers linked to lung cancer and inflammation in 24,079 adults from 16 studies. The analysis compared cotinine levels (in urine, saliva, and plasma), tobacco-specific nitrosamines (NNAL and NAT), and inflammatory markers (IL-6 and IL-1β) between smokers, e-cigarette users, non-smokers, and dual users. The findings showed that e-cigarette users had higher salivary cotinine than non-smokers, but not smokers. NNAL was significantly elevated in smokers compared to non-smokers. Salivary and plasma IL-6 and IL-1β were elevated in smokers and e-cigarette users, with plasma IL-8 significantly higher in e-cigarette users. The GRADE analysis showed low-to-high certainty of evidence and with no or moderate risk bias. The results suggest that e-cig use is associated with biomarker changes indicating inflammation and potential carcinogenesis, mirroring some effects of traditional smoking. Further longitudinal research and regulatory assessment are needed to understand the long-term health implications of e-cigarettes.
