# Supplementary material for: Inflammatory and Carcinogenic Biomarker Signatures in E-Cigarette Users: A Comprehensive Meta-Analysis of ∼24,000 Adults
Source: Int J Public Health. 2025 Nov 12;70:1608885. doi: 10.3389/ijph.2025.1608885 (PMC12646966; doi:10.3389/ijph.2025.1608885)
Supplement: Supplementary file 1 [file Supplementaryfile1.pdf]

LEGENDS TO SUPPLEMENTARY TABLES

Supplementary Table 1: Comparison of plasma interleukins

Supplementary Table 2: GRADE-based analysis for the biomarkers used in the study

Supplementary Table 3: GRADE Evidence Profile for Inflammatory Biomarkers

Supplementary Table 1

Interleukin	Parameter	non-smokers	e-cigarette	Reference
IL-1β	Mean (pg/ml)	101.9	152.7	(Singh et al. 2019;
	SD	198.7	298.3	Song et al. 2020b,
	t-value	1.026	1.024	a; Podguski et al.
	P value	0.380	0.381	2022)[10,13]
	95%CI	-214.3 to 418.1	-321.9 to 627.2	
IL-2	Mean (pg/ml)	1.53	2.05	(Song et al. 2020b,
	SD	1.93	2.89	a; Podguski et al.
	t-value	1.372	1.224	2022)[13–1]
	P value	0.304	0.346	
	95%CI	-3.27 to 6.33	-5.15 to 9.24	
IL-4	Mean (pg/ml)	1.54	1.87	(Song et al. 2020b,
	SD	2.50	3.18	a; Podguski et al.
	t-value	1.027	1.022	2022)[13–5]
	P value	0.413	0.414	
	95%CI	-4.91 to 7.99	-6.015 to 9.76	
IL-6	Mean (pg/ml)	4.98	9.48	(Singh et al. 2019;
	SD	5.18	15.1	Song et al. 2020b,
	t-value	1.923	1.256	a; Podguski et al.
	P value	0.150	0.298	2022)[10,13–15]
	95%CI	-3.26 to 13.22	-14.53 to 33.48	
IL-8	Mean (pg/ml)	17.08	18.82	(Singh et al. 2019;
	SD	8.56	14.6	Song et al. 2020b,
	t-value	3.99	2.58	a; Podguski et al.
	P value	0.028*	0.082	2022)[10,13–15]
	95%CI	3.457 to 30.69	-4.41 to 42.04	
IL-10	Mean (pg/ml)	10.51	6.613	(Singh et al. 2019;
	SD	19.68	12.27	Song et al. 2020b,
	t-value	1.069	1.078	a; Podguski et al.
	P value	0.364	0.360	2022)[10,13–15]
	95%CI	-20.8 to 41.82	-12.91 to 26.14	
IL-12	Mean (pg/ml)	2.203	2.83	(Song et al. 2020b,
	SD	3.57	4.66	a; Podguski et al.
	t-value	1.070	1.052	2022)[13–15]
	P value	0.397	0.403	

	95%CI	-6.653 to 11.06	-8.744 to 14.40	
IL-13	Mean (pg/ml)	3.74	3.47	(Song et al. 2020b, a; Podguski et al. 2022)13–15
	SD	2.8	1.82	
	t-value	2.671	3.82	
	P value	0.076	0.0316*	
	95%CI	-0.715 to 8.19	0.577 to 6.36	
IFNγ	Mean (pg/ml)	7.218	8.605	(Singh et al. 2019; Song et al. 2020b, a; Podguski et al. 2022)10,13–15
	SD	11.37	13.91	
	t-value	1.27	1.238	
	P value	0.294	0.304	
	95%CI	-10.87 to 25.31	-13.52 to 30.73	
TNFα	Mean (pg/ml)	22.13	34.57	(Singh et al. 2019; Song et al. 2020b, a; Podguski et al. 2022)10,13–15
	SD	40.63	62.69	
	t-value	1.089	1.103	
	P value	0.356	0.351	
	95%CI	-42.52 to 86.77	-65.18 to 134.3	

*statistically significant

Supplementary Table 2: GRADE-based analysis for the biomarkers used in the study

Biomarkers	Article	Risk of bias	Inconsistency	Indirectness	Imprecision	Publication bias	Certainty	Importance
**Urine - cotinine**	Bustamante (2018)	moderate (dual smoker group not included)	no inconsistency	no indirectness	not serious	not detected	high	important
	Park et al., (2019)	moderate (population size varied)	moderate (very high level seen in smokers)	no indirectness	not serious	not detected	low	important
	Goniewicz (2018)	moderate (population size variation)	no inconsistency	no indirectness	not serious	not detected	low	important
	Shahab et al., (2017)	moderate (non-smoker group not included)	no inconsistency	no indirectness	not serious	not detected	low	important
	Czoli et al., (2019)	moderate (population size variation)	moderate (significant variations)	no indirectness	not serious	not detected	high	important
**Plasma-cotinine**	Martin et al., (2016)	no risk	no inconsistency	moderate (nasal epithelial cells studied)	not serious	not detected	high	important
	Singh et al., (2019)	moderate (smoker group not included)	moderate	no indirectness	not serious	not detected	high	important
	Podguski et al., (2022)	moderate (smoker group not included)	moderate	no indirectness	not serious	not detected	high	important
**Salive cotinine**	Mokeen et al., (2018)	moderate (dual group not included)	moderate (no variation between e-cig and non-smokers)	no indirectness	not serious	not detected	high	important
	Shahab et al., (2017)	moderate (non-smokers not included)	moderate (no comparison with non-smokers)	no indirectness	not serious	not detected	high	important
	Ye et al., (2020)	no risk	no inconsistency	no indirectness	not serious	not detected	high	important
**Urine NNAL**	Goniewiez et al., (2018)	no risk	no inconsistency	no indirectness	not serious	not detected	high	important
	Shahab et al., (2017)	moderate (non-smoker group not included)	moderate inconsistency	no indirectness	not serious	not detected	high	important
	Czoli et al., (2019)	no risk	no inconsistency	no indirectness	not serious	not detected	high	important
	Holt et al., (2023)	no risk	no inconsistency	no indirectness	not serious	not detected	high	important
	Martin et al., (2016)	moderate (dual group not included)	moderate inconsistency	no indirectness	not serious	not detected	high	important

**Urine NAT**	Goniewicz et al., (2018)	no risk	moderate	no indirectness	not serious	not detected	high	important
	Shahab et al., (2017)	moderate (non-smoker not included)	no inconsistency	no indirectness	not serious	not detected	high	important
**Saliva-IL-6**	Faridoun et al., (2021)	no risk	no inconsistency	moderate (gingival status studied)	not serious	not detected	low	important
	Mokeen et al., (2018)	moderate (dual not included)	low (large variation)	moderate (periodontal status studied)	not serious	not detected	low	important
	Singh et al., (2019)	low (cig and dual group not included)	low (large variation)	no indirectness	not serious	not detected	high	important
**Saliva-IL-1b**	Faridoun et al., (2021)	no risk	no inconsistency	moderate (gingival status studied)	not serious	not detected	low	important
	Mokeem et al., (2018)	moderate (dual not included)	low (large variation)	moderate (periodontal status studied)	not serious	not detected	low	important
	Ye et al., (2020)	no risk	no inconsistency	no indirectness	not serious	not detected	high	important
	Singh et al., (2019)	low (cig and dual group not included)	low (large variation)	no indirectness	not serious	not detected	high	important

Supplementary Table 3: GRADE Evidence Profile for Inflammatory Biomarkers

Biomarkers	Article	Risk of bias	Inconsistency	Indirectness	Imprecision	Publication bias	Certainty	Importance
IL-1β	(Singh et al. 2019)	moderate (population size variation)	moderate (very large difference in results)	no indirectness	not serious	not detected	moderate	important
	(Podguski et al. 2022)	moderate (population size variation)	no inconsistency	no indirectness	not serious	not detected	high	important
	(Song et al. 2020b)	moderate (population size variation)	no inconsistency	no indirectness	not serious	not detected	high	important
	(Song et al. 2020a)	moderate (population size variation)	no inconsistency	no indirectness	not serious	not detected	high	important
IL-2	(Podguski et al. 2022)	moderate (population size variation)	no inconsistency	no indirectness	not serious	not detected	high	important
	(Song et al. 2020b)	moderate (population size variation)	no inconsistency	no indirectness	not serious	not detected	high	important
	(Song et al. 2020a)	moderate (population size variation)	no inconsistency	no indirectness	not serious	not detected	high	important
IL-4	(Podguski et al. 2022)	moderate (population size variation)	no inconsistency	no indirectness	not serious	not detected	moderate	important
	(Song et al. 2020b)	moderate (population size variation)	no inconsistency	no indirectness	not serious	not detected	high	important
	(Song et al. 2020a)	moderate (population size variation)	no inconsistency	no indirectness	not serious	not detected	high	important
IL-6	(Singh et al. 2019)	moderate (population size variation)	no inconsistency					
	(Podguski et al. 2022)	moderate (population size variation)	moderate (very large difference in results)	no indirectness	not serious	not detected	moderate	important

	(Song et al. 2020b)	moderate (population size variation)	no inconsistency	no indirectness	not serious	not detected	high	important
	(Song et al. 2020a)	moderate (population size variation)	no inconsistency	no indirectness	not serious	not detected	high	important
IL-8	(Singh et al. 2019)	moderate (population size variation)	moderate (very large difference in results)	no indirectness	not serious	not detected	moderate	important
	(Podguski et al. 2022)	moderate (population size variation)	no inconsistency	no indirectness	not serious	not detected	high	important
	(Song et al. 2020b)	moderate (population size variation)	no inconsistency	no indirectness	not serious	not detected	high	important
	(Song et al. 2020a)	moderate (population size variation)	moderate (very large difference in results)	no indirectness	not serious	not detected	high	important
IL-10	(Singh et al. 2019)	moderate (population size variation)	moderate (very large difference in results)	no indirectness	not serious	not detected	moderate	important
	(Podguski et al. 2022)	moderate (population size variation)	no inconsistency	no indirectness	not serious	not detected	high	important
	(Song et al. 2020b)	moderate (population size variation)	no inconsistency	no indirectness	not serious	not detected	high	important
	(Song et al. 2020a)	moderate (population size variation)	no inconsistency	no indirectness	not serious	not detected	high	important
IL-12p70	(Podguski et al. 2022)	moderate (population size variation)	no inconsistency	no indirectness	not serious	not detected	high	important
	(Song et al. 2020b)	moderate (population size variation)	no inconsistency	no indirectness	not serious	not detected	high	important
	(Song et al. 2020a)	moderate (population size variation)	moderate (very large difference in results)	no indirectness	not serious	not detected	high	important

IL-13	(Singh et al. 2019)	moderate (population size variation)	moderate (very large difference in results)	no indirectness	not serious	not detected	moderate	important
	(Podguski et al. 2022)	moderate (population size variation)	no inconsistency	no indirectness	not serious	not detected	high	important
	(Song et al. 2020b)	moderate (population size variation)	no inconsistency	no indirectness	not serious	not detected	high	important
	(Song et al. 2020a)	moderate (population size variation)	moderate (very large difference in results)	no indirectness	not serious	not detected	high	important
IFN-γ	(Singh et al. 2019)	moderate (population size variation)	moderate (very large difference in results)	no indirectness	not serious	not detected	moderate	important
	(Podguski et al. 2022)	moderate (population size variation)	no inconsistency	no indirectness	not serious	not detected	high	important
	(Song et al. 2020b)	moderate (population size variation)	no inconsistency	no indirectness	not serious	not detected	high	important
	(Song et al. 2020a)	moderate (population size variation)	moderate (very large difference in results)	no indirectness	not serious	not detected	high	important
TNF-α	(Singh et al. 2019)	moderate (population size variation)	moderate (very large difference in results)	no indirectness	not serious	not detected	moderate	important
	(Podguski et al. 2022)	moderate (population size variation)	no inconsistency	no indirectness	not serious	not detected	high	important
	(Song et al. 2020b)	moderate (population size variation)	no inconsistency	no indirectness	not serious	not detected	high	important
	(Song et al. 2020a)	moderate (population size variation)	no inconsistency	no indirectness	not serious	not detected	high	important
